# Differences in self-reported disruptions in mental health treatment during COVID-19 in a national household sample: impact of severity of functional impairment

**DOI:** 10.21203/rs.3.rs-4676128/v1

**Published:** 2024-07-29

**Authors:** Margaret Wang, Scott Graupensperger, mark olfson, Natalie Bareis, mark edlund, Maria Monroe-DeVita, Ronald Kessler, Mackenzie Tennison, Katherine Winans, Lydia Chwastiak

**Affiliations:** University of Washington; University of Washington; Columbia University; University of Washington; University of Washington; University of Washington; Harvard University; University of Washington; University of Washington; University of Washington

**Keywords:** Mental health access, serious mental illness, COVID-19, mental health disparities

## Abstract

**Objective:**

This report uses data from Mental Disorders Prevalence Study (MDPS), a large epidemiologic study that provided national prevalence estimates of seven mental disorders based on the Structured Clinical Interview for DSM-5 (SCID), to assess the odds of treatment disruption during COVID for SMI and non-SMI groups.

**Methods:**

This cross-sectional study conducted from 2020 to 2022 included 2,810 household participants with any lifetime mental health treatment. Weighted logistic regressions estimated the odds of reporting disruptions in access to mental health care or psychotropic prescriptions due to COVID. SMI was broadly defined as having an MDP diagnosis and serious functional impairment (GAF ≤50, a validated and widely used cutoff). Non-SMI groups were a mental diagnosis without serious impairment (MDPS diagnosis, GAF >50) and any lifetime treatment and no serious impairment (no MDPS diagnosis, GAF >50).

**Results:**

The SMI and mental disorder without serious impairment groups had approximately 6.4- and 2.4-greater odds, respectively, of reporting inability to access mental health care and 4- and 3- greater odds, respectively, of having prescriptions delayed, relative to the group with any lifetime treatment. Among those with serious mental illness, having Medicare insurance increased the odds of reporting inability to access mental health care.

**Conclusions:**

Individuals with SMI were much more likely to experience treatment disruptions throughout the pandemic than non-SMI groups.

## Introduction

The mental health treatment gap, the proportion of people with measured or perceived need for mental health care that does not receive treatment, has been recognized as a major public health challenge for more than twenty years [1]. The COVID-19 pandemic (COVID) exacerbated disparities in access to healthcare and represents an opportunity to understand who are the most vulnerable in a population. This is especially important for individuals with serious mental illness (SMI), who often experience poverty, unstable housing, social isolation and cognitive deficits that make them vulnerable to care disruptions [2, 3]. While the definition of SMI varies [4, 5], the U.S. Department of Health and Human Services (DHHS) in 1992 defined SMI as any mental health condition that cause severe/serious daily functional impairment and this is one of the definitions of SMI currently used by the American Psychiatric Association [6, 7]. To our knowledge, no studies have examined the impact of COVID on mental health treatment for individuals meeting the DHHS criteria for SMI using a large, national community sample of individuals, to include those not in care and with diagnostic interviews by clinicians.

Studies on the impact of COVID and access to mental health treatment among individuals with SMI have mainly utilized insurance claims data or focused on a particular health system using electronic health records, which limits generalizability of findings [8, 9, 10, 11, 12]. The literature shows conflicting results, which could be explained by the varying definitions of SMI across studies as well as different study populations, and past studies mainly compared the impact of COVID in 2020 to pre-pandemic cohorts. Additional research beyond early pandemic (2020) and broadening SMI to include individuals with a broader range of mental disorders that limited function, would expand our understanding of the magnitude of the mental health treatment gap among individuals with SMI. Going beyond a single state or insurance database and using a comprehensive definition of SMI would allow for better understanding of treatment disparities.

This study utilized a national household sample of US adults aged 18–65 (*N* = 4,764) from the Mental Disorders Prevalence Study (MDPS), a large epidemiologic study in which trained clinicians administered the Structured Clinical Interview for DSM-5 (SCID) [13] to diagnose seven mental disorders and five substance use disorders. The MDPS provide clinical diagnostic accuracy using semi-structured DSM-5 based interviews, a national sample, and data collection occurring throughout the two years of COVID (2020–2022). We defined SMI as meeting past-year criteria for an MDPS mental health diagnosis and evidence of severe functional impact based on a Global Assessment of Functioning (GAF) score ≤ 50 in the past year [14]. We compared the group with SMI to two other groups of MDPS participants: those with a mental disorder but without serious impairment (those meeting criteria for a past year MDPS mental disorder that causes moderate-to-mild functional impairment (i.e., GAF > 50); and those reporting any lifetime mental health treatment but without serious impairment (those not meeting criteria for a past year MDPS mental disorder and moderate-to-mild functional impairment). The study investigated across the three groups the prevalence of 1) inability to access mental health treatment due to COVID or 2) mental health prescriptions delayed due to COVID and 3) identified sociodemographic factors associated with inability to access mental health treatment during COVID among those with SMI.

## Methods

### Data source

The MDPS was a national survey funded by the US Substance Abuse and Mental Health Services Administration (SAMHSA) designed to identify past 12-month prevalence estimates of seven mental health disorders and five substance use disorders and rates of treatment in the United States [15]. Data collection occurred between October 2020 and October 2022. MDPS included adults aged 18–65 from household and non-household (prisons, state psychiatric hospitals, and homeless shelters) samples. Here, we only present data from the household sample, who comprise approximately 90% of the U.S population, as mental health access in non-household samples (e.g. psychiatric hospitals) does not reflect that of the general population.

A three-stage sampling design was used to select participants for the household sample. First, randomly sampled US addresses were sent an invitation to participate in the survey (N = 234,270) and up to 2 individuals (age 18–65) per household were randomly selected to complete a screening interview.

Second, participants completed a screener (N = 29,048) that enabled categorization according to evidence of psychiatric symptoms to oversample individuals who may have a mental disorder. Third, 12,906 screener respondents were selected for a DSM – 5 based clinical interview, of which 4,764 adults completed. Survey weights were used to adjust the oversampling back to a national representative estimate [16]. Details about the sampling design and other methods have been published previously [15, 16].

### Mental disorders measurements

Trained clinicians administered the Structured Clinical Interview for DSM-5 (SCID5) [^13^] via videoconference or telephone to diagnose of one of seven past 12-month mental disorders: major depressive disorder, generalized anxiety disorder, bipolar 1 disorder, schizophrenia spectrum disorders (schizophrenia, schizoaffective disorder, and schizophreniform disorder), post-traumatic stress disorder, obsessive compulsive disorder, and anorexia nervosa. Level of mental health functioning was rated by the trained clinicians using the Global Assessment of Functioning (GAF) scale at the time of the interview [14]. The GAF rates mental illness severity based on symptoms and a global measure of psychological, social, and occupational functioning and does not include functional impairment due to physical or environmental limitations [17]. It is scored from 0 to 100 with lower scores indicating more symptoms or functional impairment (the lowest symptoms or functioning score is recorded as the overall GAF score). A score of 50 or lower has been validated and widely used to reflect serious functional impairment or serious symptoms [14, 18].

Any MDPS substance use disorder refers to a past-year SCID diagnosis of mild, moderate or severe alcohol use disorder, opioid use disorder, stimulant use disorder, sedative/ hypnotic/ anxiolytic use disorder, or cannabis use disorder.

### Reported Mental health access and impact during COVID

The 4,764 participants who completed the clinical interview were asked if they had “ever received professional counseling, medication or other treatment to help with mental health, emotions, or behavior.” Only the participants who answered yes (n = 2,810), indicating any lifetime history of mental health treatment, were asked four questions about the impact of COVID-19 on mental health treatment. Two of these questions “were you unable to access mental health care due to COVID-19” and “did you have mental health prescriptions delayed due to COVID-19” are the focus of these analyses. Responses were binary (Yes/No”). The analytic sample for the present study is the 2,810 participants with lifetime history of mental health treatment.

### Study populations

The present analyses do not include anorexia nervosa because of the very low prevalence. “Serious mental illness” was defined as having any of the other six MDPS diagnoses and a GAF score ≤ 50. The second group, “*MDPS* mental disorder without serious impairment,” was defined as having any of the six MDPS diagnosis and a GAF score > 50. The final group, “any lifetime mental health treatment without serious impairment,” was defined as those in the household sample without an MDPS diagnosis and GAF score > 50. This last group includes those without a mental illness and would also capture those who have a mental disorder that was not one of the seven MDPS diagnoses (e.g., panic disorder).

### Clinical and sociodemographic characteristics and treatment utilization

Clinical characteristics included MDPS mental health diagnoses, any substance use disorder (at least one of the five MDPS substance use disorders), and GAF scores.

Utilization of mental health treatment was also assessed, including receipt of outpatient mental health treatment in the past year, number of outpatient mental health visits in the past year, and receipt of prescription medication for a mental disorder in the past year.

Sociodemographic characteristics included age, birth sex, race, Hispanic ethnicity, marital status, education level, rural or urban locality, household income, and insurance type, which we categorized as five ordered mutually exclusive ordered categories of private, Medicare, Medicaid, other (VA or other military), and no insurance.

### Statistical analysis

Descriptive statistics were used to characterize clinical and sociodemographic characteristic of the three groups (serious mental illness, mental disorder without serious impairment, and any lifetime treatment without serious impairment). Estimates were weighted to account for oversampling of individuals with elevated risk of schizophrenia spectrum or other mental health disorders in the MDPS design and reflect the general population. We calculated the weighted prevalences of inability to access mental health treatment and delayed mental health prescriptions and adjusted for sex, age, ethnicity, marital status, rural status, income, number of outpatient visits and prescription medications, and any of the five MDPS substance use disorder in the past year.

We used weighted logistic regressions to estimate the odds of being unable to access mental health care due to COVID-19 (model 1) and having mental health prescriptions delayed due to COVID-19 (model 2), comparing the SMI group and mental disorder without serious impairment to the group with any lifetime treatment and no serious impairment. Model 2 included only participants who endorsed receiving a prescription for a mental health disorder in the past year (n = 1,794). The models adjusted for sociodemographic characteristics, as well as the date of the clinical interview, which was calculated as months since the beginning of the COVID-19 pandemic (March 2020). We examined the SMI group (n = 368) to identify risk/protective factors associated with inability to access mental health treatment, which was again done using weighted logistic regression.

Analyses were conducted in R using the ‘survey’ package to account for the complex sampling design.^19^ The study was approved by the Advarra Institutional Review Board.

## Results

### Clinical and sociodemographic characteristics

The SMI group had a mean GAF score of 43 (SD = 0.9), which corresponds to serious impairment functioning (e.g., no friends, unable to keep a job) or serious symptoms; the mental disorder without serious impairment had a mean GAF score of 64 (SD = 0.6), indicating moderate impairment in functioning or moderate symptoms; and the group with any lifetime treatment and no serious impairment had a mean GAF score of 77 (SD = 0.7), which corresponds to transient and expected reactions to psychosocial stressors if symptoms are present and no more than slight impairment in functioning (Table 1).

Among the 368 adults with SMI, 17.0% had a schizophrenia spectrum diagnosis compared to 1.5% in the mental disorder without serious impairment group (n = 1,112). The prevalences of bipolar I, post-traumatic stress disorder, and OCD were two to three times as high in the SMI group compared to the mental disorder without serious impairment group. The majority of those with SMI (79.1%) and mental disorder without serious impairment (77.1%) and over half of the any lifetime treatment without serious impairment group (54.6%) received outpatient mental health care in the past year. The prevalence of any substance use disorder ranged from a high of 27.1% for those with serious mental illness to 9.2% for those with any lifetime treatment and no current serious symptoms. The mean number of outpatient mental health visits was 21.0 (SD = 2.4) in the past year for those with SMI, 13.4 (SD = 1.5) for those with a mental disorder without serious impairment and 6.5 for the any lifetime treatment without serious impairment group. Over 70% of those with SMI received at least one mental health prescription over the past year compared to 67.4% of the mental disorder without serious impairment group and 48.0% the group with any lifetime treatment without serious impairment.

### Reported COVID-related inability to access mental health care or prescription delay

**Figure 1:**
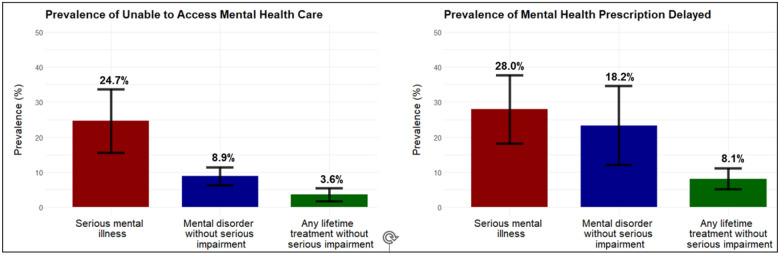
Weighted prevalence estimates of mental health care disruptions due to COVID-19 stratified by mental health category. The first plot (left) entails the full sample (*N* = 2,810), and the second plot (right) entails only the subset of participants who endorsed having a prescription in the past year (*n* = 1794). ***Note***: Adjusted for sex, age, ethnicity, marital status, rural status, income, number of outpatient visits and prescription medications, and any of the five MDPS substance use disorder in the past year

As shown in Fig. 1, the weighted prevalence of reported inability to access mental health care during COVID was highest for those with SMI (24.7%) and lowest for the any lifetime treatment group (3.6%). Similarly, the prevalence of reporting mental health prescription delay ranged from a high of 28.0% for those with SMI to 8.1% for those with any lifetime treatment. The models in Table 2 show that the SMI and mental disorder without serious impairment groups had approximately 6.4- and 2.4- greater odds, respectively, of reporting inability to access mental health care and 4- and 3- greater odds, respectively, of having prescriptions delayed, relative to the group with any lifetime treatment without serious impairment. The timing of the interview (time since the onset of the pandemic in March 2020) did not have a significant impact on either outcome. Having a household income of $75,000 or greater and having other/military insurance significantly decreased the odds of reporting inability to access care, while having any substance use disorder significantly increased the odds of reporting inability to access mental health care and of having prescriptions delayed.

### Characteristics of adults with SMI reporting inability to access mental health care

Among the subset of participants with SMI, those with Medicare insurance were 4 times greater odds of reporting inability to access mental health care than those with private insurance (Table 4). For the 364 individuals under 65 with SMI, around 20% (n = 74) were Medicare enrollees. Younger age was also associated with increased odds of inability to access care, as was being currently married relative to never married.

## Discussion

In this national household sample of 2,810 U.S. adults who had received mental health treatment at some point in their lives, the impact of COVID on mental health treatment access was compared across three groups: participants with SMI, a mental disorder without serious impairment, and those with any lifetime treatment without current serious impairment. The SMI group had substantially increased odds of reporting inability to access mental health care and of having psychotropic prescription delays relative to those with any lifetime mental health treatment. Participants with a mental disorder without current serious impairment also had increased odds of these treatment barriers compared to those with any lifetime treatment, though the odds were attenuated compared to the SMI group. Among individuals with SMI, having Medicare insurance increased the odds of reporting inability to access care during COVID.

Compared to the any lifetime treatment group, individuals with SMI had 6 times greater odds of reporting an inability to access mental health care. Those with a mental disorder without serious impairment also had an increased likelihood of disruption of treatment compared to those with any lifetime treatment, though this risk was attenuated compared to the SMI group. These results suggest that individuals experiencing greater functional impairment from a mental disorder had elevated risks of not receiving adequate treatment during COVID.

The lack of a consensus definition of SMI makes comparing our findings and those of previous studies challenging, but our findings are in line with previous research that reported reduced access to mental health care for individuals with SMI during early COVID (2022) compared to pre-COVID who were not able to access telemedicine services [20, 12, 21, 10]. Our results are in contrast to a Veteran’s Administration (VA) study that found fewer telemedicine video visits, but no difference in overall visits, in the initial COVID period compared to the pre-pandemic period, which may reflect the unique VA health system [9]. A New Hampshire Medicaid study of community mental health centers that defined *any* mental illness as SMI found a 4.9% increase in telemedicine interruptions compared to the year prior, and that patients with schizophrenia had the least interruptions, but it is unclear if these findings would generalize to populations and states that includes large urban centers [11].

Our study extends previous work showing that early in the pandemic, among Medicare beneficiaries, there were decreases in psychiatric prescription fills for individuals with SMI even with telehealth [8], and the expansion of telemedicine did not increase medication adherence in this population [22]. Individuals with SMI had a four-fold greater odds of reporting having prescriptions delayed than those with any lifetime treatment. Medicare beneficiaries, in general, tend to be older and more likely to have a disability compared to the general population. Our results extend the psychotropic prescription disruption findings to a broader SMI population of housed non-elderly adults and suggest that the disruptions persisted past the early pandemic (2020). Regardless of mental illness group, having any substance use disorder increased the odds of disruptions in accessing mental health care and delays in psychotropic prescription access compared to no substance use disorder. We did not directly examine access to substance use treatment, but previous research shows individuals with substance use disorders tend to have low rates of substance use treatment and engagement [23].

Among the SMI subpopulation, those with Medicare compared to private insurance were more likely to report inability to access mental health care. One explanation is that non-elderly adults on Medicare (20% of SMI sample) need to have a disability to enroll in Medicare, and thus, require a high level of treatment access that in turn may contribute to their risk of experiencing an inability to access mental health care. Alternatively, the barriers to mental health services for Medicare beneficiaries, such as inaccurate provider directories and insurance denials [24], or providers less likely to take public than private insurance [25], could explain the findings. Further research is needed to clarify if individuals with SMI covered by Medicare during COVID had a provider who did not have enough availability to meet their needs, or whether they had difficulty finding a provider.

This study has several implications. First, individuals with SMI had the highest odds of reporting inability to access mental health care or having prescriptions delayed. Despite being a group in high need of ongoing treatment access, this suggests that the access received still might not match the needs of this group. Regardless of diagnosis, individuals with a MDPS mental disorder causing serious functional impairment (the SMI group) had the highest odds of unmet treatment needs. Second, among those with SMI, those with Medicare compared to private insurance were significantly more likely to report inability to access mental health care. More research will be needed to understand whether those with Medicare insurance face barriers such as shortage of providers who accept Medicare [25, 26], inaccurate provider directories [24], or insurance denials [27], or whether the provided services are not enough to meet their needs.

A key strength of the study that increases the importance of the findings is the study design. Mental disorders were diagnosed based on semi-structured psychiatric interviews conducted by trained clinicians, which increases diagnostic accuracy. The survey was based on a national household probability sample and included a large number of participants and is not limited to a single database or population. We included data beyond the initial months of the pandemic. Trained clinicians provided assessments of severity of functional impairment in addition to diagnoses.

Among study weaknesses, inability to access mental health treatment was a binary response that did not capture key service nuances such as quality of care, number of times of occurrence, duration of inability to access treatment and was not confirmed by pharmacy or health utilization data. Nevertheless, self-report healthcare utilization has been shown to correlate with administrative data [28]. The survey question did not distinguish between in-person versus telemedicine access and prior research showed heterogeneity between access and type of service delivery [12]. The study could not determine whether inability to access care was due to establishing care, barriers to existing care or subjective dissatisfaction with the number or quality of care received. The lifetime mental treatment group could have not needed to access mental health care, which may contribute to them reporting having no access difficulties. Though a national sample, the overall MDPS survey response rate was low, which could reduce the representativeness of the sample [15]. Weighting, however, may attenuate selection bias. Third, the MDPS study included only seven mental disorders, and many individuals who did not meet criteria for an MDPS disorder may have needed treatment for a different mental disorder (e.g., panic disorder, personality disorders) [29]. The GAF is a scoring system of mental illness severity based on global function but does not specify diagnoses and scoring can be subjective [17]. In this study, clinicians were trained in GAF administration and had team case reviews to reach consensus. Future studies of impairment should also include functional outcomes, such as employment status or social functioning. How to characterize function in those with mental illness is not yet defined or standardized, and our findings should be taken within this context.

In conclusion, in this large national survey, conducted 2020–2022, individuals with SMI were more likely to report mental health treatment disruptions (inability to access mental health care, delayed psychotropic prescription) during COVID, compared to non-SMI groups. Individuals with SMI on Medicare were more likely to report inability to access care. To narrow the mental health treatment gap, future research should also focus on discerning which individuals with a mental illness causing serious functional impact are most likely to experience access difficulties and mechanisms for access difficulties. More research should discern whether mental health services meet the needs of those whose illness has an associated serious impact on daily life.

## Figures and Tables

**Table 1:** Clinical and sociodemographic characteristics and treatment utilization

	Serious mental illness^1^ (n = 368)		Mental disorder without serious impairment^2^ (n = 1,112)		Any lifetime treatment without serious impairment^3^ (n = 1,330)	
	Raw	Weighted [95% CI]	Raw	Weighted [95% CI]	Raw	Weighted [95% CI]
MDPS Diagnoses						
Schizophrenia Spectrum	13.6%	17.0% [9.4, 24.6]	1.3%	1.5% [0.1, 2.9]	---	---
Major Depression	62.5%	57.1% [47.1, 67.1]	66.0%	65.1% [56.5, 73.7]	---	---
Generalized Anxiety Disorder	48.8%	41.5% [30.9, 52.1]	48.3%	44.8% [37.4, 52.2]	---	---
Bipolar I	12.8%	12.1% [4.3, 19.9]	6.1%	4.9% [3.1, 6.7]	---	---
Post-Traumatic Stress Disorder	30.1%	34.6% [25.2, 44.0]	15.6%	14.1% [9.6, 18.6]	---	---
Obsessive Compulsive Disorder	16.9%	19.9% [11.9, 27.9]	10.6%	10.4% [5.7, 15.1]	---	---
Received any outpatient mental health care (past year)	85.3%%	79.1% [70.1, 88.1]	77.6%	77.1% [71.2, 83.0]	58.1%	54.6% [49.1, 60.1]
Mean outpatient visits (past year)	26.4	21.0 [16.3, 25.6]	14.3	13.4 [10.4, 16.3]	8.2	6.5 [5.0, 8.0]
Received prescription medications for mental health (past year)	82.6%	73.5% [64.7, 82.3]	67.5%	67.4% [60.9, 73.9]	55.8%	48.0% [42.3, 53.7]
Mean GAF Score	42.3	43.0 [41.3, 44.7]	64.0	63.9 [62.8, 65.0]	75.8	77.0 [75.6, 78.5]
Mean age	39.9	38.4 [36.0, 40.7]	38.2	35.3 [33.3, 40.7]	43.9	42.0 [40.7, 43.5]
Female	68.8%	65.2% [56.6, 73.8]	72.8%	64.7% [57.8, 71.6]	64.1%	55.5% [49.6, 61.4]
Race						
White	74.9%	69.4% [60.0, 78.8]	78.5%	77.5% [71.0, 84.0]	82.4%	77.7% [71.5, 83.9]
Black	12.7%	17.6% [9.1, 26.1]	8.6%	8.6% [5.3, 11.9]	8.7%	12.9% [7.4, 18.5]
Asian	2.2%	3.9% [0.0, 8.2]	4.8%	3.3% [1.0, 5.5]	3.8%	2.7% [1.2, 4.3]
Other	10.2%	9.0% [3.7, 14.4]	8.1%	10.6% [5.3, 15.9]	5.1%	6.6% [3.2, 10.1]
Hispanic Ethnicity	15.2%	10.2% [6.7, 13.7]	13.6%	10.7% [6.8, 14.5]	11.3%	13.9% [7.8, 19.9]
Rural/non-urban	14.7%	15.9% [7.9, 23.9]	13.5%	23.6% [13.4, 33.8]	14.7%	18.7% [11.4, 26.0]
Marital status						
Now Married	23.9%	28.6% [18.5, 38.8]	36.3%	36.8% [28.5, 45.0]	46.9%	51.7% [45.2, 58.2]
Divorced/widowed/separated	25.0%	20.2% [12.8, 27.6]	17.7%	15.2% [11.0, 19.5]	19.7%	17.4% [12.3, 22.5]
Never married	51.1%	51.2% [40.6, 61.8]	46.0%	48.0% [39.0, 57.0]	33.4%	30.9% [26.0, 35.9]
Education level						
Less than High School	6.0%	14.2% [5.5, 22.9]	2.5%	6.6% [1.9, 11.3]	2.6%	6.4% [2.9, 10.0]
High School	47.4%	53.0% [42.3, 63.6]	31.1%	45.7% [37.7, 53.8]	27.9%	44.4% [38.5, 50.4]
College Degree	35.4%	29.6% [20.8, 38.5]	41.9%	38.6% [29.4, 47.8]	38.9%	32.9% [28.0, 37.8]
Graduate or Professional	11.2%	3.2% [1.6, 4.7]	24.5%	9.1% [6.5, 11.7]	30.5%	16.2% [12.7, 19.7]
Household Income						
< $20,000	34.5%	35.4% [24.3, 46.5]	17.1%	19.0% [14.2, 23.9]	12.2%	16.5% [11.2, 21.8]
$20,000 – $49,9999	31.8%	35.9% [25.6, 46.3]	25.0%	30.2% [21.9, 38.5]	19.8%	20.3% [16.2, 24.3]
$50,000 – $74,999	13.6%	9.8% [6.0, 13.7]	16.7%	15.6% [11.6, 19.6]	17.0%	17.8% [12.2, 23.4]
$75,000 or greater	20.1%	18.8% [11.3, 26.3]	41.2%	35.2% [27.1, 43.3]	51.0%	45.4% [39.6, 51.3]
Insurance						
Private Insurance	40.8%	31.6% [22.4, 40.8]	69.0%	61.6% [54.3, 68.9]	73.8%	61.4% [54.5, 68.3]
Medicare	23.3%	21.6% [13.8, 29.4]	7.5%	9.4% [5.7, 13.1]	9.7%	13.8% [8.7, 18.9]
Medicaid	40.5%	51.9% [41.5, 62.3]	21.4%	25.3% [19.6, 31.0]	15.3%	25.2% [19.1, 31.3]
Military or Other	12.8%	11.5% [6.6, 16.4]	6.2%	8.5% [3.8, 13.2]	6.1%	7.4% [4.1, 10.7]
No Insurance	5.2%	3.9% [1.9, 5.9]	5.1%	6.1% [3.4, 8.8]	5.3%	7.7% [4.8, 10.6]
Any MDPS SUD (past year)	30.2%	27.1% [18.4, 35.8]	17.2%	20.6% [12.5, 28.8]	9.9%	9.2% [6.8%. 11.6]

**Note**: 2,810 adults in the MDPS household sample endorsed lifetime mental health treatment and were included in the analyses.

1. Any MDPS diagnosis and GAF score ≤ 50;

2. Any MDPS diagnosis and GAF score > 50;

3. No past-year MDPS diagnosis and GAF score > 50.

SUD = Substance use disorder.

**Table 2:** Associations between treatment access or delayed prescriptions and mental health category

	Unable to Access Mental Health Care (n = 2,810)^1^			Delayed Mental Health Prescriptions (n = 1,794)^2^		
	Adjusted Odds Ratio	95% CI	*p*-value	Adjusted Odds Ratio	95% CI	*p*-value
Intercept	0.05	[0.01, 0.35]	0.003	0.13	[0.03, 0.66]	0.015
Mental Health Category (Ref = No Past-Year MDPS Diagnosis)						
Serious Mental Illness	6.37	[3.16, 12.84]	< .001	3.66	[1.92, 6.97]	< .001
Mental disorder without serious impairment	2.35	[1.19, 4.63]	.015	2.96	[1.79, 4.88]	< .001
Age	0.99	[0.97, 1.02]	.541	0.99	[0.97, 1.01]	.241
Birth Sex (Ref = male)	1.28	[0.76, 2.14]	.350	1.07	[0.62, 1.83]	.817
Race (Ref = White)						
Black	0.94	[0.36, 2.42]	.890	0.57	[0.29, 1.15]	.115
Asian	0.53	[0.19, 1.48]	.223	0.52	[0.14, 1.98]	.336
Other	0.89	[0.46, 1.73]	.734	0.25	[0.10, 0.64]	.005
Hispanic (Ref = not Hispanic)	2.32	[0.98, 5.46]	.055	1.13	[0.61, 2.12]	.695
Marital Status (Ref = Currently Married)						
Divorced/Widowed/Separated	0.63	[0.28, 1.43]	.267	0.94	[0.44, 2.01]	.872
Never Married	0.64	[0.33, 1.24]	.183	0.83	[0.41, 1.67]	.594
Rural (Ref = Not rural)	0.50	[0.24, 1.05]	.066	1.22	[0.61, 2.45]	.570
Household Income (Ref < $20,000)						
$20,000 – $49,9999	0.68	[0.33, 1.41]	.290	1.52	[0.74, 3.12]	.253
$50,000 – $74,999	0.40	[0.14, 1.12]	.081	1.26	[0.63, 2.55]	.507
$75,000 or greater	0.39	[0.16, 0.93]	.034	0.65	[0.30, 1.41]	.274
Insurance Type (Ref = Private Insurance)						
Medicare	1.50	[0.58, 3.84]	.397	0.64	[0.27, 1.50]	.296
Medicaid	0.97	[0.41, 2.28]	.936	1.01	[0.48, 2.13]	.979
Other or Military	0.34	[0.13, 0.88]	.027	0.70	[0.29, 1.70]	.422
No Insurance	0.70	[0.25, 1.91]	.476	0.60	[0.20, 1.84]	.370
Any MDPS SUD (past year)	2.51	[1.26, 5.00]	.010	2.76	[1.52, 5.04]	.001
Interview Date (Months since March 2020)	1.03	[0.97, 1.08]	.338	1.00	[0.97, 1.04]	.861

**Note:** Weighted logistic regression models were used to estimate associations.

1. The model included all adults who received lifetime mental health care

2. The model used the subset of participants who endorsed having a prescription in the past year

SUD = substance use disorder.

**Table 3:** Associations with sociodemographic characteristics and treatment access among adults with serious mental illness

	Unable to Access Mental Health Care (n = 368)		
	Adjusted Odds Ratio	95% CI	*p*-value
Intercept	28.03	[2.45, 320.12]	.008
Age	0.93	[0.90, 0.96]	< .001
Birth Sex (0 = Male, 1 = Female)	0.69	[0.36, 1.31]	.253
Race (Ref = White)			
Black	0.47	[0.15, 1.50]	.199
Asian	0.06	[0.01, 0.72]	.026
Other	1.14	[0.41, 3.15]	.797
Hispanic (0 = No, 1 = Yes)	1.96	[0.60, 6.36]	.258
Marital Status (Ref = Currently Married)			
Divorced/Widowed/Separated	0.72	[0.23, 2.33]	.584
Never Married	0.27	[0.09, 0.75]	.013
Rural (0 = No, 1 = Yes)	0.46	[0.08, 2.61]	.377
Household Income (Ref < $20,000)			
$20,000 – $49,9999	0.97	[0.31, 3.08]	.959
$50,000 – $74,999	0.32	[0.05, 2.00]	.220
$75,000 or greater	0.48	[0.13, 1.73]	.257
Insurance Type (Ref = Private Insurance)			
Medicare	3.87	[1.07, 14.05]	.040
Medicaid	0.65	[0.21, 2.03]	.450
Other or Military	0.24	[0.05, 1.09]	.063
No Insurance	1.10	[0.30, 4.04]	.884
Any MDPS SUD (past year)	1.48	[0.53, 4.18]	.451
Interview Date (Months since March 2020)	0.97	[0.91, 1.03]	.318

**Note:** Weighted logistic regression models were used to estimate associations

SUD = substance use disorder.

## Data Availability

https://www.icpsr.umich.edu/web/ICPSR/studies/38953
